# A Text-Book of Pharmacology

**Published:** 1902-03

**Authors:** 


A Text-Book of Pharmacology. Including Therapeutics, Materia Medica, Pharmacy, Prescription writing, Toxicology, etc. By Torald Sollmann, M. D., Assistant professor of Pharmacology and Materia Medica, Western Reserve University, Cleveland, Ohio. Royal octavo volume of 880 pages, fully illustrated. Philadelphia and London: W. B. Saunders & Co., 1901. Cloth, $3.75 net.

This excellent work is by far the best of its kind which has yet appeared. Its author, by basing the study of therapeutics on a systematic knowledge of the nature and properties of drugs, emphasizes the close relation between pharmacology and practical medicine. Physicians, students, druggists, in short, everyone interested in the use of medicine, will find a study of the work a very great help. It is not a book that is likely to lie idle on one's shelf. R.

